# Editorial: Genetic, Environmental and Synergistic Gene-Environment Contributions to Craniofacial Defects

**DOI:** 10.3389/fcell.2022.887051

**Published:** 2022-03-24

**Authors:** Peter J. Anderson, Karen J. Liu, Mary L. Marazita, Sebastian Dworkin

**Affiliations:** ^1^ Australian Craniofacial Unit, Women’s and Children’s Hospital, Adelaide, SA, Australia; ^2^ Faculty of Health Sciences, University of Adelaide, Adelaide, SA, Australia; ^3^ Nanjing Medical University, Nanjing, China; ^4^ Centre for Craniofacial and Regenerative Biology, King’s College London, London, United Kingdom; ^5^ Department of Oral Biology, School of Dental Medicine, Center for Craniofacial and Dental Genetics, University of Pittsburgh, Pittsburgh, PA, United States; ^6^ Department of Human Genetics, Graduate School of Public Health, University of Pittsburgh, Pittsburgh, PA, United States; ^7^ Department of Psychiatry and Clinical and Translational Science Institute, School of Medicine, University of Pittsburgh, Pittsburgh, PA, United States; ^8^ Department of Physiology, Anatomy and Microbiology, La Trobe University, Melbourne, VIC, Australia

**Keywords:** craniofacial, gene-environment interaction, genetic, cleft palate, craniosynostosis

Of the defects that affect formation of the craniofacial skeleton (the skull, face and jaws of vertebrates), the most common are those that affect fusion of the lip and secondary (hard) palate (resulting in cleft lip/palate) (Dixon et al., 2011), and those that lead to premature fusion of the bones of the skull (craniosynostosis). As a research and clinical field, we know that both genetic mutations (Wilkie and Morriss-Kay, 2001; Twigg and Wilkie, 2015; Weinberg et al., 2018) and fetal exposure to environmental toxins (Brent, 2004) individually may lead to craniofacial defects (CFD), although, in general, the synergistic nature of genetic predisposition to CFD and maternal toxin intake remains largely understudied.

In general, there are two approaches for identifying novel Gene-Environment Interactions (GxE). One approach utilises large-scale big data analyses at the population level; these can unearth both genetic polymorphisms and differences in genetic background (e.g., ethnicity), to identify risk-factors associated with increased susceptibility to craniofacial defects (Marazita, 2012; Leslie and Marazita, 2013). Often, these population studies may be further combined with understanding relevant societal and behavioural risk-factors, such as national living standards, socioeconomic background, intake of alcohol, drugs and tobacco smoke and environmental pollutants. Combining such datasets allows strong GxE risk factors for CFD to be identified (Dixon et al., 2011).

The second approach relies on utilising animal models harbouring defined genetic mutations that lead to congenital abnormalities during embryogenesis (Liu, 2016). By exposing these animal models to bioactive compounds (under carefully controlled supplementation regimes), novel compounds may be discovered that either exacerbate or reduce the incidence and penetrance of those defects (Greene and Copp, 2005).

Both approaches have yielded substantial advances, and understanding these GxE is critical for patient healthcare. Prospective parents carrying sensitising genetic mutations or polymorphisms can be advised to avoid certain foods, alcohol, medications etc., to minimise the risk of exacerbating an otherwise mild defect in their offspring, resulting in a much healthier start to life for these babies. Moreover, understanding how to overcome defects of genetic origin (that would otherwise lead to severe CFD) provides even more far-reaching possibilities—dramatically reducing the need for extensive, expensive and frequently inaccessible post-natal surgical intervention and post-operative care.

This special issue of 10 articles (comprising eight original studies and two reviews) explores some exciting advances in understanding GxE in the aetiology of craniofacial defects, utilising *in vitro*, *in silico*, zebrafish and mouse models as well as employing Genome-Wide Association Studies (GWAS) and human genetic approaches to obtain population-level data from disparate patient cohorts and datasets.

Firstly, this issue comprises two excellent reviews on factors that affect the fetal environment *in utero*, and consequences of exposure to both exogenous and endogenous stressors on craniofacial development, particularly on the fidelity, survival and functional differentiation of neural crest cells (NCCs). Fitriasari and Trainor explore the features of neural crest cells that make them uniquely sensitive to replication stress. They review the link between maternal diabetes, oxidative stress, genotoxic damage and the resultant death of NCCs as a means by which the penetrance and phenotypic variability of various craniofacial disorders is determined. Sánchez et al. review the emerging link between anti-depressant use during pregnancy (typically those medications used to maintain or increase levels of serotonin) and the incidence of craniofacial disorders. Together these reviews highlight the importance of maternal health and well-being during pregnancy as an important contributing factor to normal embryonic and fetal craniofacial development.

Secondly, we present four excellent studies centring on the rich data to be mined from human genetic approaches, GWAS and patient trios, in understanding GxE more broadly at the population level. Carlson et al. show a significant association between genetic predisposition of patients with certain Single Nucleotide Polymorphisms (SNPs) near the genes VGLL2 and PRL with adverse outcomes on craniofacial development following maternal exposure to alcohol and smoking respectively. Zhang et al. utilised two large databases of case-parent trios (comprising over 3,300 patients) to investigate GxE in patients of diverse ethnic backgrounds, and similarly identified novel risk loci following exposure to smoking, alcohol and multivitamin supplementation. The group of Mukhopadhyay et al. utilised the extensive resource of the Pittsburgh Orofacial Clefts Multiethnic study, comprising ∼12,000 individuals, to determine the statistical power in identifying and characterising common, disparate and novel risk loci predisposing to orofacial clefting based on individual ancestry. In a complementary approach, Machado et al. used whole exome sequencing (WES) to identify putative polymorphisms in genetic protein-coding regions associated with non-syndromic oral clefts in a multiethnic Brazilian population, and found significant associations between cleft lip and palate in the folic-acid associated metabolism genes LRP6 and methyltransferase (MTR).

These studies elegantly highlight the rich potential of GWAS and WES to not only identify novel genetic risk factors, but importantly, to also apply these genetic discoveries to identifying novel GxE following *in utero* toxin exposure. The emerging applications of big data to identifying biologically relevant risk-factors present an extremely promising approach to unravel novel deleterious GxE in the manifestation of CFD in newborns.

Finally, the development of novel, non-human, experimental models for increasing our understanding of craniofacial disorders is also paramount. Narumi et al. explore the relationship between chemical exposure and the *Wnt*-signalling pathway, using the excellent and highly-tractable zebrafish embryo model as a screening tool for investigating novel GxE. Johnson et al. report an elegant *in vitro* assay to model interaction between epithelial and mesenchymal tissue and the chemical modulation of *Shh*-gradients, providing a novel tool to investigate the complex cross-talk that occurs between these tissues during development, classically, in the context of the developing hard palate. Cross et al. have developed a ground-breaking new computational model to predict the clinical outcomes (head and skull shape) following reconstructive surgery for craniosynostosis , and this interesting study may serve as a strong foundation for further extensive studies. We look forward with anticipation to see if the outcomes of this sophisticated approach will positively impact on future patient healthcare. Lastly, the group of Yoshioka et al. explore the relationship between microRNA signalling, cell proliferation, and all-trans retinoic acid (*at*RA), elegantly demonstrating that inhibiting miR-124-3p activity in mice rescues cleft palate caused by *at*RA exposure. Together, these studies present novel findings from non-human approaches, highlighting the utility and conservation of animal and predictive models to enhance our understanding of craniofacial defects.

Ultimately, the identification and investigation of novel genes identified from patient datasets, together with functional characterisation in animal models presents the best combined strategy to rapidly increase our understanding of GxE in the context of the aetiology of craniofacial defects.
